# Not just a goiter: Uncovering primary thyroid lymphoma

**DOI:** 10.6026/973206300214039

**Published:** 2025-11-15

**Authors:** Venkata Kaumudi Ayaluri, Angel Jose, Harshitha Reddy, Arooba Khurram

**Affiliations:** 1 Department of Accident and Emergency, Clinical Fellow, Bronglais General Hospital, Aberystwyth, Wales, United Kingdom; 2 Department of Vascular Surgery, Junior Clinical Fellow, St Thomas Hospital, London, United Kingdom; 3 Department of Medicine and Critical Care, Consultant, Navjeevan Hospital, Hyderabad, India

**Keywords:** Primary thyroid lymphoma, diffuse large B-cell lymphoma, hashimoto's thyroiditis, thyroid mass, R-CHOP

## Abstract

Primary thyroid lymphoma (PTL) is a rare, aggressive neoplasm of the thyroid gland that occurs most frequently in older women. We describe a 60-year-old woman who presented to our hospital with a primarily painless, progressively enlarging neck swelling over the previous four months. Imaging showed an enlarged thyroid gland and mass effect in the surrounding neck soft tissue. Fine needle aspiration revealed a predominance of lymphoid cells to suggest that this hypothesized mass was a thyroid neoplasm. Biopsy of the thyroid gland confirmed non-Hodgkin lymphoma of the thyroid. She was treated with standard CHOP chemotherapy. This case highlights the conundrum of PTL in a rapidly enlarging mass of the thyroid gland and the need to address this early with better diagnosis and therapeutic plan.

## Background:

Thyroid lymphoma is an uncommon tumor, representing only 1-5% of all thyroid cancers and less than 2% of extranodal lymphomas [1]. Aging females are most commonly affected and the disease has a strong association with Hashimoto's thyroiditis, in which chronic autoimmune inflammation is thought to create a permissive microenvironment for lymphomagenesis [2]. Histological variants of thyroid lymphoma include diffuse large B-cell lymphoma (DLBCL) and mucosa-associated lymphoid tissue (MALT) lymphoma, which are the most commonly described. Clinical presentation of primary thyroid lymphoma (PTL) is often as rapidly enlarging neck mass, usually with compressive symptoms such as dysphagia, dyspnea or hoarseness [3]. These features can mimic anaplastic thyroid carcinoma or subacute thyroiditis, complicating timely diagnosis. Ultrasound and CT may show a hypoechoic (i.e. dark) thyroid mass with homogeneous or heterogeneous echotexture and pseudo-infiltrative pattern [4]. Unfortunately, FNA is often non-diagnostic and final diagnosis requires histopathological and immunohistochemical analysis of a core needle or surgical biopsy [5]. The management of patients with thyroid lymphoma is largely based on histological subtype and stage. Chemotherapy and radiotherapy are the mainstay of treatment. Prognosis for localized MALT lymphoma is generally favorable, while prognosis is worse for advanced disease with DLBCL histology [6]. Therefore, it is of interest to describe cases of thyroid lymphoma to highlight its diagnostic challenges and emphasize the importance of clinicopathological correlation in distinguishing PTL from other thyroid malignancies.

## Case presentation:

A 60-year-old female housewife presented to the outpatient department with a complaint of swelling in the lower neck for 2 months. The swelling was insidious in onset, approximately the size of a marble, which had not progressed in size. The patient reported no associated symptoms such as pain, fever, difficulty swallowing, or changes in voice. Her medical history included hypertension managed with Atenolol (25 mg) for two years. There was no history of radiation exposure or significant systemic illnesses such as tuberculosis or diabetes mellitus. Family history was unremarkable. Upon examination, the patient was conscious, coherent and well-oriented. She appeared moderately built and nourished with no signs of pallor, icterus, cyanosis, clubbing, or generalized lymphadenopathy. On further examination of the neck a solitary firm swelling was noted in the anterior triangle of the lower neck on the left side, measuring 2.5 x 1.5 cm. The swelling moved with deglutition and had well-defined borders. There was no local rise in temperature or tenderness and no palpable lymph nodes in the neck. Table 1 (see PDF) summarizes the hematological and biochemical parameters of the patient in comparison with normal reference ranges. The results show mild anemia (hemoglobin 11.2 g/dl, hematocrit 26.3%) with microcytic, hypochromic indices (low MCV, MCH and MCHC), suggesting iron deficiency or anemia of chronic disease. Leukocyte and platelet counts were within normal limits, while the erythrocyte sedimentation rate (32 mm/hr) was elevated, indicating underlying inflammation. Renal and liver function tests were normal, though serum urea appeared unusually low and may reflect either a lab reporting error or dietary variation. Thyroid function tests were within the reference range, indicating euthyroid status despite the thyroid mass. Fine Needle Aspiration Cytology of the swelling showed a colloid background with sheets of thyrocytes and predominant lymphoid cells (Figure 1 - see PDF). Under general anesthesia in supine position through a collar incision, left hemithyroidectomy was performed with preservation and exploration of both recurrent laryngeal nerves and all parathyroid glands (Figure 2 - see PDF). The surgery was uneventful. Upon Biopsy of the sample sent after the Left Hemi-Thyroidectomy Microscopic examination revealed colloid-filled follicles lined by cuboidal epithelium with dense lymphoid aggregates and monomorphic lymphoid cells (Figure 3 - see PDF). Following it Immunohistochemistry was done and it demonstrated CD3 positivity (T-cells) and CD20 positivity (B-cells) (Figure 4 - see PDF, 5 - see PDF, 6 - see PDF). All these investigations lead to the clinical diagnosis as primary thyroid lymphoma, possibly non-Hodgkin lymphoma.

## Discussion:

Primary malignant lymphoma of the thyroid is uncommon, comprising less than 2% of thyroid malignancies. It typically presents as a rapidly growing mass and predominantly affects elderly females in their sixth decade of life, with a female-to-male ratio of approximately 4:1 [1]. Patients may present with symptoms such as hoarseness, dyspnea, dysphagia and pain. Primary thyroid lymphomas constitute about 2.5-7% of all extra nodal lymphomas [1]. There are two distinct clinical and prognostic groups within thyroid lymphoma, with diffuse large B-cell lymphoma (DLBCL) being the most common subtype, accounting for up to 70% of cases. The majority of primary thyroid lymphomas are B-cell lymphomas associated with Epstein-Barr virus and Hashimoto's thyroiditis [2]. Epidemiological evidence and indirect molecular findings from studies on immunoglobulin heavy chain variable region (IGHV) genes suggest that chronic persistent antigen stimulation may lead to malignant transformation, constituting a significant pathogenic mechanism for thyroid lymphoma. It is important to note that even experienced pathologists may find it challenging to distinguish primary thyroid lymphoma from thyroiditis [7]. Histologically, a key diagnostic feature is the packing of the follicular lumen by lymphoid cells, which is not present in thyroiditis. Immunohistochemistry has significantly improved the accuracy of cytological diagnoses of lymphoma by enabling the specification of lineage and developmental stages [8]. The presence of antibodies against B-cell antigens such as CD19 and CD20 identifies B-cell lineage among lymphoid cells. Additionally, staining for Kappa (K) and Lambda (X) light chains can help identify abnormal clonal populations. While CD10 is typically present in most follicular lymphomas, it is generally negative in both DLBCL and MALT lymphoma. Most DLBCL cases are Bcl-6-positive and approximately half are Bcl-2 positive [9]. In MALT lymphoma, the presence of immunoglobulin light chains and Bcl-2 IgM heavy chain staining in plasma cell components serves as additional diagnostic clues [10]. The prognosis for thyroid lymphoma largely depends on the histopathological type and stage at diagnosis. Treatment options typically include chemotherapy and radiation therapy, while surgical intervention may be necessary for some patients to relieve local pressure symptoms. Early diagnosis and appropriate treatment are crucial for achieving favorable outcomes in patients with primary thyroid lymphoma [11].

## Conclusion:

Primary thyroid lymphoma is a rare but treatable malignancy that requires early recognition for optimal outcomes. Accurate histological diagnosis and timely systemic therapy are crucial, especially for aggressive subtypes. A multidisciplinary approach ensures effective management and improved patient prognosis.
